# Reinforcement of the Framework for Experiential Education in Healthcare in Serbia: Post-Implementation Project Review within Pharmacy Education

**DOI:** 10.3390/pharmacy7030092

**Published:** 2019-07-15

**Authors:** Marina Odalović, Jelena Parojčić, Dragana Vasiljević, Danijela Đukić Ćosić, Ljiljana Tasić

**Affiliations:** Faculty of Pharmacy, University of Belgrade, Vojvode Stepe 450, 11221 Belgrade, Serbia

**Keywords:** experiential education, interprofessional education, teaching competency development, pharmacy education

## Abstract

**Background:** The Erasmus+ project “Reinforcement of the Framework for Experiential Education in Healthcare in Serbia” (ReFEEHS) has been undertaken with the aim to: (i) reinforce and modernize experiential education (ExEd) in the health sciences curricula, (ii) introduce interprofessional education (IPE), and (iii) promote teaching competency development of academic staff and teacher practitioners/clinician educators. The aim of this paper is a post-implementation review of the project activities and outcomes with the emphasis on the impact and sustainability in pharmacy education. **Methods:** Project Logical framework matrix has been employed as planning, monitoring and evaluation tool which summarizes the main project objectives, project outcomes, relevant activities, indicators of progress, sources of verification, assumptions and risks. **Results:** The key project outcomes are: (i) update of competency-based curricula and development of quality assurance framework for students professional practice placements; (ii) development and introduction of interprofessional teaching and learning activities through joint curriculum delivery; and (iii) development and implementation of Teaching Certificate in Health Professions Education (TCinHPE) study program. The short-term impact of project activities and outcomes has been assessed based on the feedback received from relevant stakeholders, as well as self-evaluation of participants enrolled in new/updated curricula. Sustainability of project results is necessary in order to achieve long-term impact envisioned as increased level of professional competency of health science students; increased level of teaching competency of academic staff and teacher practitioners; improved patient healthcare and harmonisation with the EU practice and policies. **Conclusions:** The project outcomes contributed to building capacity at the Serbian universities involved in terms of collaboration between the healthcare professions and, in curriculum and academic staff development. It is expected that improved curricula will positively impact professional competency development of pharmacy students, graduates employability and increased workforce mobility. Meeting the quality standards of the European Higher Education Area will contribute to visibility of Serbian universities and their internationalisation, which is one of the strategic aims of improvement.

## 1. Introduction: Project Rationale

Contemporary health professions education (HPE) is determined by the increasingly rapid knowledge growth in biomedical sciences, regulatory requirements for health professions and demand for experiential and interprofessional teaching and learning activities as the foundation for future interprofessional collaborative practice in patient centered healthcare. It is also recognised that educational reforms must address the health needs of their communities.

The term “experiential education” (ExEd) is used to denote supervised structured or semi-structured teaching and learning activities that take place in a practice setting and involves real-life situations and inter-personal interactions with patients, caregivers, and other health professionals (it may also be referred as practice-based learning, clinical experience, or students professional practice placement) [1].

Interprofessional education (IPE) is defined as an educational approach when two or more professions learn with, from and about each other to improve collaboration and the quality of care [2]. The benefits of interprofessional collaborative practice in healthcare have been well recognized [3,4,5]. In 2010, WHO published the Framework for Action on Interprofessional Education & Collaborative Practice [5]. More recently, the European Healthcare Students’ Associations (EHSAS) united to consider the important issues of interprofessional collaboration and multidisciplinary approaches in health professions education [6].

As regulated professions which are subject to mutual recognition of qualifications between the EU member states, health professions need to be compliant with specific and rigorous professional standards at both the national and international level. General standards and requirements are specified in the EU Directive 2013/55, as well as a number of documents issued by relevant national and international authorities and professional bodies [7]. The status of regulated profession brings additional responsibility to higher education institutions (HEI) and relevant national regulatory bodies. EU Directive 2013/55 provides the general requirement for clinical experience of health professions to be integrated part of undergraduate studies. This request has been only partially addressed in the health sciences curricula at the Republic of Serbia (RS) universities, with the situation being the most critical in pharmacy education where professional practice placements were organized mainly in the pre-registration year following graduation from a five-year didactic curriculum.

In order to become compliant with contemporary EU practice, there was an urgent need to review health sciences curricula, reinforce ExEd and introduce IPE activities at RS universities. Reinforcement of integrated, competency-based curriculum, implementation of active learning, evidence-based teaching and assessment, use of information technology, shared educational resources, emphasis on ExEd and IPE activities have been recognized as issues of principal interest in health professions education in RS. To implement advanced teaching and learning practices, building faculty capacity has been necessary with regards to improvements needed at the institutional and regulatory level, and teaching competency development of both academic staff and teacher practitioners/clinical educators. The project entitled “Reinforcement of the Framework for Experiential Education in Healthcare in Serbia” (ReFEEHS), was proposed and accepted for co-financing by the Education, Audiovisual and Culture Executive Agency within the program Erasmus + Key Action 2 Capacity Building in Higher Education (https://refeehs.com/). The aim of the present paper is a post-implementation review of the project activities and outcomes with the emphasis on the impact and sustainability in pharmacy education.

## 2. Materials and Methods: Project Description

### 2.1. Project Objectives

Main ReFEEHS project objectives have been defined as:

(1) Reinforcement and modernization of experiential education in health sciences curricula (Medicine, Pharmacy, Dentistry and Nursing);
(2) Introduction of interprofessional teaching and learning activities, including new joint interprofessional courses for all health sciences students;
(3) Teaching competency development of academic staff and teacher practitioners/clinician educators, including introduction of Teaching Certificate in Health Professions Education (TCinHPE) study programme.

### 2.2. Project Consortium

The ReFEEHS Consortium involved eight higher education institutions: four RS universities (University of Belgrade-project coordinator, University of Kragujevac, University of Novi Sad, University of Niš) and four EU universities (Trinity College Dublin, Medical University Sofia, University of Lisbon-Faculty of Pharmacy, University of Pecs).

### 2.3. Project Activities

Core ReFEEHS project activities were focused on the accomplishment of the main project objectives through: (i) identification of competency-based outcomes related to ExEd, IPE and TCinHPE curricula, (ii) development of relevant educational content and resources, and (iii) establishment of quality assurance measures and procedures, as well as continuous evaluation of the program (Appendix A). Institutional capacity building of the RS HEIs was based on academic staff training and joint development of educational resources and quality assurance documents with the EU partner institutions. During a three years period of time (from 15 October 2015 to 14 October 2018), thematic workshops, panel discussions and a number of working group meetings were organized at each of the RS partner institutions. Structured study visits to the four EU partner institutions provided an opportunity for academic exchange of experience and practices in health professions education (https://refeehs.com/). A number of individual RS academic staff exchanges with EU partner institutions were organized with the individual tasks and opportunities available for job shadowing and participation in relevant educational activities throughout the project’s lifetime. A project website was established in the early preparation phase and served as a repository of information for all the interested parties, and also as a communication and management platform for consortium partner institutions (https://refeehs.com/). Additionally, in order to develop the TCinHPE, collaboration with the health professions from each of the RS Universities and the Faculty of Philosophy of the University of Belgrade was undertaken. The quality of the implemented activities and reached outcomes was assured by continuous monitoring and assessment according to the proposed indicators of progress defined within the Project Logical framework matrix. Both internal evaluation trough Quality working group activities, as well as the external evaluation performed by the appointed External expert, were employed. Joint efforts were also directed towards dissemination and exploitation of project outcomes and their sustainability.

### 2.4. Competency Evaluation

With the purpose of ExEd competencies and teaching competency evaluation, relevant self-evaluation questionnaires with accompanied lists of appropriate competencies were developed. A four point Likert scale was used in evaluation of ExEd competencies, with the following answers: 1—I have theoretical knowledge about the competence; 2—I have theoretical knowledge about the competence, but I can not carry out the competence related task; 3—I have theoretical knowledge about the competence and I am capable to carry out the competence related task under the supervision of the clinician educator; 4—I have theoretical knowledge about the competence and I am able to carry out the competence related task independently. Self-evaluation of teaching competency employed five point Likert scale with the following possible answers: 1—not competent; 2—not competent enough; 3—moderately competent; 4—very competent; 5—fully competent. ExEd and teaching competency, were evaluated twice, at the course beginning, and after the course completion.

## 3. Results: Project Outcomes

The ReFEEHS Project Logical framework matrix was employed as planning, monitoring and evaluation tool which summarizes the main project objectives, project outcomes, relevant activities, indicators of progress, sources of verification, assumptions and risks (Appendix A).

At the beginning of the project, the ReFEEHS consortium conducted a comprehensive survey on the attitudes of healthcare sciences students, and academic staff and practitioners related to ExEd, IPE and teaching competency development at the Universities of Belgrade, Kragujevac, Niš and of Novi Sad, with the support of the professional chambers, the professional associations and the healthcare institutions. The results were publicized in The Need for Change report and have provided the baseline against which improvements were measured [8].

### 3.1. Reinforcement and Modernization of Experiential Education

The main project achievement related to reinforcement and modernization of ExEd is reflected in the joint efforts of the four RS universities and each of the health professions to establish common set of quality standards for students professional practice placements. Guideline document defining relevant expectations and quality standards related to: (i) educational content, (ii) competency-based outcomes, (iii) teaching, learning and student assessment methodology, (iv) placement sites and clinician educators, and (v) student responsibilities have been prepared and published [9]. Furthermore, educational resources and equipment necessary to support students preparedness for professional practice placements have been provided and included simulated training in the pharmacy skills lab. Substantial efforts have been directed towards development of the e-platform for students ExEd management and administration (http://147.91.1.76/login/index.php). The platform is designed as an information system which includes: (i) students, placement sites and practice supervisors databases, (ii) documents management and archiving, (iii) student practice scheduling/practice site allocation, (iv) student progress monitoring, (v) student, supervisor and practice site evaluation, (vi) quality assurance of practice learning, and (vii) effective communication among all the user groups. Beside administrative support, this information system supports students e-portfolio, and assessment of students activities in the form of on-line tests/essays.

The framework developed for ExEd within pharmacy education involved the development of a pharmacies network as certified placement sites, and pharmacy professionals engaged as clinician educators. Initial training of clinician educators and continuous support from the responsible academic departments has been provided. Although certain differences in the level of competency achievement were notable among pharmacy students at three RS universities, the most prominent improvements have been observed with regards to patient counselling, health promotion and disease prevention at the University of Belgrade (2.03 vs. 3.38, before and after ExEd curriculum delivery, respectively), prescription evaluation with regards to administrative and medication errors at the University of Novi Sad (1.75 vs. 3.12, before and after ExEd curriculum delivery, respectively), and patient counselling with regards to drug waste management at the University of Kragujevac (1.81 vs. 3.32, before and after ExEd curriculum delivery, respectively) (Table 1).

### 3.2. Development of Interprofessional Teaching and Learning Course

Interprofessional teaching and learning activities have been introduced in the health sciences curricula (Medicine, Pharmacy, Dentistry and Nursing) in the form of new joint elective course at each of the RS participating universities. The new course has been adopted by the relevant university authorities and included in the study programs IPE course has been designed as modular blended learning program delivered through three “face-to-face” sessions accompanied by relevant e-learning contents and tasks. Common curricula and shared educational resources (online course and course handbook) have been developed and implemented (Table 2) [10]. Details related to development of IPE course are presented in Table 2.

Interprofessional modules were linked to the relevant competency-based outcomes for each health sciences profession. Joint interprofessional courses were implemented at a pilot scale for the selected groups of students during the third project year (2017–2018) with the contribution of academic staff from all the health sciences study programs. Students from different study programs within the same University attended all classes together and worked in small groups. The number of participants in IPE pilot course presented in Table 3.

### 3.3. Teaching Competency Development of Academic Staff and Clinician Educators

It has been recognized that in order to progress through educational reform, highly skilled and motivated teaching staff is essential. The ReFEEHS project provided an opportunity for a group of junior academic staff from all RS partner institutions to enroll in external distance learning postgraduate courses in health professions education. They have been recruited to develop the TCinHPE course for academic staff and clinician educators and serve as tutors to their peers. They are expected to take over the leadership in further educational reforms and act as the agents of change. TCinHPE has been approved by the Senate of the University of Belgrade as a continuing professional development program for academic staff and clinician educators engaged in health professions education. It was developed and delivered by a multi-disciplinary group with international participation including representatives of each health profession and pedagogues from the University of Belgrade, as well as representatives from the Trinity College Dublin. TCinHPE is delivered as blended learning course including both online and face-to-face teaching and learning activities, with the “contact” teaching and learning sessions based on “learning by doing”, and different small group active teaching activities. Apart of e-learning content developed, the Teaching & Learning in Health Professions Education–Guide for academic staff has been published which includes the recommendations on the Policy for teaching competency development and evaluation as an important aspect of academic staff appointment and promotion [11]. The first cohort of 38 academic staff and teacher practitioners has successfully completed TCinHPE in the third project year, 2018. Teaching competency self-evaluation performed before and after completing the program indicated that notable improvement has been achieved with regards to: (i) curriculum evaluation and self-evaluation, (ii) integration of pedagogical and professional knowledge and skills in everyday teaching practice, (iii) students’ assessment and (iv) curriculum development. Improvement of teaching and learning and enhancement of study programs based on collaboration between different health sciences faculties were seen as the most important potential benefits of the program. The second call for application has been released in February 2019, and current generation of 31 participants is engaged in the TCinHPE.

### 3.4. Overall Project Impact

An equivalent of 450 academic staff members and clinician educators have directly benefited from the ReFEEHS training courses. Four thematic workshops have been organized under the following topics: “Current practice and challenges in education within the health professions”, “Current practice and challenges in interprofessional education of healthcare professionals”, “Competency based outcomes in healthcare professions education”, and “Contemporary aspects of quality assurance in health professions education” (https://refeehs.com/events-2/). Four structured study visits to EU participating universities, external online courses in health professions education, specific training of teaching staff involved in delivery of the new IPE course, workshops for clinician educators/practice supervisors and the new TCinHPE were also different modes of teacher training provided through the ReFEEHS project in order to prepare teaching staff for new roles and innovative teaching and learning methodologies. The majority of the training events held in RS were accredited as continuing education courses for health professionals. A total of around 2500 final year health sciences students, among which 600 pharmacy students, have been engaged in the updated ExEd program. ReFEEHS project activities and outputs have been presented to wider professional and general public through a number of meetings presentations, including ReFEEHS symposia and open-days, articles published, the project website and mass media including the national broadcasting agency (https://refeehs.com).

## 4. Discussion: Project Impact and Sustainability

ReFEEHS project introduced innovative concepts in health professions education in line with the most progressive global recommendations promoting: (1) quality ExEd, (2) interprofessional education of health sciences students, and (3) a formal study program for teaching competency development of academic staff and clinician educators engaged in health professions education. Innovative teaching and learning methodology, based on active learning supported by digital technology in the form of blended learning courses has been employed.

ReFEEHS project impact on the institutional level can be described by: (i) students engagement in the new/updated curricula, (ii) increased teaching competency of academic staff and teacher practitioners, (iii) increased level of collaboration with healthcare institutions and healthcare practitioners acting as students practice supervisors, (iv) development of ExEd quality assurance framework, (v) increased students competency and level of preparedness for practice, (vi) introduction of active teaching and learning methodology, (vii) educational resources developed and purchased.

The ReFEEHS project impact on the national and regional level will be facilitated by the availability of educational resources and examples of best practice which should help in development of similar initiatives and modernization of health professions education at other higher education institutions in the country and abroad. The ReFEEHS project outcomes contribute to further reform of higher education in line with the emerging demands and expectations related to competence development, recognition of qualifications, workplace learning and digital skills. It will support re-examination of the existing institutional policies and amendments and updating of institutional and national regulation. This could be accomplished via a review of the impact of the existing accreditation standards and promotion of their evolution in line with contemporary EU practice, including updating of the existing teaching competency performance indicators in university and faculty regulation on academic staff promotion. They also contribute to harmonization with EU practices, policies and regulation in health professions education.

Impact and sustainability of the project outcomes with regards to the innovation in teaching, learning and student assessment will be monitored and evaluated by the faculty management and curriculum committee through regular annual evaluation, and periodic self-evaluation. Improved students competency will be measured based on the results of licensure exam and feedback received from supervisors of the pre-registration internship year. Long term impact on improved patient care and patient health outcomes will be measured through regular monitoring of healthcare system performance conducted by the national Institute of Public Health and other relevant institutions.

Sustainability of the project outcomes is supported by their institutionalization as part of the formal curricula and existing HEI regulation. It is envisioned that the reinforced ExEd will be extended to six-month students’ professional practice which will replace the mandatory pre-registration internship of health professionals, bringing to higher education institutions increased responsibility for professionalism, competency development and readiness for practice of future graduates. It is also envisioned that the IPE course will advance as obligatory subject for all health sciences students, supported by the relevant quality standards for health professions education. ReFEEHS TCinHPE is the first professional development programme for academic staff and clinician educators in the region. It is envisioned that it will attract interest also of health professions educators from abroad. Admission of international applicants will promote further academic exchange and contribute to the objectives and initiatives identified across the European Higher Education Area. Best practices and experiences exchanged within the ReFEEHS consortium, including shared educational resources and quality standards, represent a base for further collaboration and continuous quality improvement. The successful outcomes were achieved within each profession and between all of the RS Universities as a consequence of collaboration and co-operation. The development of these relationships and the scale and scope of the shared achievements facilitated by the ReFEEHS project will help to sustain its impacts. However, continuous efforts and intensive communication are necessary in order to secure further support from the academic and professional community and to overcome the resistance to change.

## 5. Strengths and Limitations of the Study

Evaluation of the competency reached by attendees within ExEd course and TCinHPE was based on self-evaluation. The biggest disadvantage of this type of evaluation is related to objectivity issues. It needs reflection on personal strengths and weaknesses, which can result in over- or underestimation of both. Accordingly, competency self-evaluation could be observed as the limitation of the project results evaluation. However, self-evaluation is the first essential step in any evaluation process. It helps to consider past performance to set future targets, and it can provide information useful for planning and improvement which could be observed as advantage of employed methodology within competency evaluation in this project [12]. The above mentioned results have to be interpreted with regards to denoted strengths and limitations.

## 6. Conclusions

Post-implementation review of the ReFEEHS project activities and outcomes indicates that substantial the impact in pharmacy education has been achieved. Support received from EACEA facilitated and accelerated educational reforms in line with contemporary standards and expectations in health professions education. Raising awareness on the necessity of educational reform and of the importance of quality teaching and learning in pharmacy education, new/updated courses and educational resources developed should be emphasized as the most important short-term impacts. Long-term impacts will be achieved through further exploitation leading to improved professional competency development of pharmacy students, graduates employability and subsequently, quality of patient health care. The improved curriculum, teaching and learning, meeting the quality standards of the European Higher Education Area will contribute to visibility of Serbian universities and their internationalisation, which is one of the strategic aims of improvement.

## Figures and Tables

**Table 1:** Pharmacy students’ competency evaluation before and after six-week professional practice placement.

COMPETENCY	UB-FPh		UKg-FMS		UNS-FM	
	Before (N = 35)	After (N = 55)	Before (N = 80)	After (N = 80)	Before (N = 65)	After (N = 66)
Patients health needs assessment	2.40	2.95	2.14	3.10	1.69	2.50
Patient counselling on disease prevention and health promotion	2.03	3.38	2.13	3.17	1.60	2.58
Patient counselling on rational drug use	2.89	3.45	1.82	3.30	2.18	2.41
Proper and adequate choice of dosage form. dose and package	2.46	3.24	1.77	3.12	1.63	2.74
Recording. assessment and prevention of drug interactions	2.14	2.91	2.69	3.52	2.28	2.82
Selection of equipment. procedures and substances needed for medicine compounding	2.89	3.24	2.49	3.27	1.72	2.70
Appropriate drug dispensing	2.80	3.53	2.24	3.84	1.69	2.71
Prescription checking and evaluation	3.03	3.56	2.19	3.17	1.75	3.12
Appropriate medical device dispensing	2.31	2.76	2.11	3.20	1.61	2.29
Identifying. documenting. and addressing drug-related problems	2.17	2.73	2.17	3.30	1.71	2.41
Adequate labelling of medicinal product. providing necessary oral and written information	3.00	3.53	2.26	3.39	1.94	2.39
Patient counselling on storage conditions. expiration date and the disposal of unused medicines	3.34	3.64	1.81	3.32	1.78	2.58

Abbreviations: UB-FPh: University of Belgrade, Faculty of Pharmacy; UKg-FMS: University of Kragujevac, Faculty of Health Sciences; UNS-FM: University of Novi Sad, Faculty of Medicine. Likert scale points: 1—I have theoretical knowledge about the competence; 2—I have theoretical knowledge about the competence, but I cannot carry out the competence related task; 3—I have theoretical knowledge about the competence and I am capable to carry out the competence related task under the supervision of the clinician educator; 4—I have theoretical knowledge about the competence and I am able to carry out the competence related task independently.

**Table 2:** IPE course development (curricula, teaching and learning methods, learning resources).

**Course Content**
*General*
IPE (term and importance, experiences from other countries, evaluation). Teamwork skills. Collaborative practice. IPE and collaborative practice competency.
*Special parts*
Acute coronary syndrome (etiology, clinical signs and symptoms, care). Diabetes (etiology, clinical signs and symptoms, medical treatment). Geriatrics (aging characteristics, special features og geriatric population, basic geriatric syndrome, polypharmacy, pharmacotherapy in geriatric population, pharmaceutical forms for geriatric population).
**Tutorials**
Case studies: *Acute coronary syndrome patient, Diabetic patient; Geriatric patient*
Analysis and discussion of care plan designe for students of all involved profesions. Care plan was presented from different points of view (medical, dentist, nurse, pharmacist)
**Methods of Teaching and Learning**
Online lectures; on site work in small groups for case discussion
**Student’ Evaluation and Assessment**
Formative on-line assessments (on-line test and essays)
Summative assessment-on line essay for patient case study
**Students Eligible for Enrolment:** Students in the Last Two Year of the Study Programs
**Enrolment of Teachers/Facilitators**
Clinical assistants, professor, professional experts, who completed the workshop for IPE educators
**Literature—Course Handbook**
The “Interprofessional education” handbook has been published in October 2018 (10).
The handbook consists of three main chapters:
(1) Interprofessional education—basis of the collaborative practice,
(2) Team work and communication skills—basis of the efficiently collaborative practice and
(3) Collaborative practice—basis for the quality of health care

**Table 3:** Participants in IPE pilot course.

Institution	Students	Teachers
University of Belgrade	60	16
University of Novi Sad	15	4
University of Niš	40	2
University of Kragujevac	12	4
Total	136	26

## Appendix A

**Table A1:** ReFEEHS Project Logical Framework Matrix.

**Erasmus+ CBHE Project Reinforcement of the Framework for Experiential Education in Healthcare in Serbia–Logical Framework Matrix**			
**Overall Objective**	**Indicators of Progress**	**Sources of Verification**	
Modernisation and quality assurance of experiential education of health sciences students in Serbia aimed at the improved patient healthcare and harmonisation with the EU practice and policies (incl. EU Directive 2013/55)	Increased level of professional competency of health science students (in Medicine, Dentistry, Pharmacy and Nursing) Increased level of teaching competency of academic staff and clinician educators Improved employability of health sciences graduates Harmonization with EU practice and policies	- Reports on the students and teaching staff competency assessments - Feedback from the National Institute of Public Health, employers and other stakeholders - Relevant EU/WHO evaluation reports	
**Specific Project Objectives**	**Indicators of Progress**	**Sources of Verification**	**Assumptions and Risks**
**Experiential education (ExEd) framework development** including competency based learning outcomes, relevant quality assurance documents and procedures; **Introduction of interprofessional education (IPE)** as a foundation for health care delivery based on interprofessional collaborative practice; **Teaching competency development (TCD) of academic staff and clinician educators** including development of Teaching Certificate in Health Professionals Education (HPE) continuing professional development course;	- ExEd curriculum upgrades and updates approved at all RS HEIs and implemented in the third project year - QA documents for ExEd management and administration designed and approved - IPE curriculum designed and approved at all RS HEIs and implemented in the third project year - Policy for teaching competency development and evaluation publicised - Teaching Certificate in HPE approved and implemented in the third project year	- Decisions/approvals of relevant university/faculty authorities - Amendment of relevant policies and regulations - Students, teacher practitioners and course coordinators feedback - Policy for teaching competency development and evaluation adopted by relevant university/governmental authorities	- Support from governmental authorities, professional chambers and associations, and students associations; - Partner institutions staff motivated and interested to participate and timely complete the project activities; - Motivation of health professionals to be included in ExEd as clinician educators and their commitment to learning and teaching - Complex and time-consuming procedures for regulatory amendments;
**Outputs & Outcomes**	**Indicators of Progress**	**Sources of Verification**	**Assumptions and Risks**
**(P) Experiential Education in Health Professions: Needs for Change Report**	- ReFEEHS Needs for Change Report publicized - upgraded educational outcomes for all study programmes involved in the project introduced in Course catalogues at all the RS HEIs - new educational contents and resources developed main QA documents developed and adopted including relevant standards, guidelines and code of practice - E-platform User Requirements Specification defined - practical placement sites and teacher practitioners’ databases developed - ExEd E-platform designed - equipment purchased and distributed to RS HEIs - Teaching Certificate in HPE educational contents and resources developed - policy for TCDE adopted - all final year students (approx. 2500) attend the upgraded EE curricula - 136 students at all RS HEIs completed Pilot IPE courses - E-platform adaptation at each HEI and mapping of experiential curriculum - 11 RS academic staff completed external courses in Health Professionals Education and engaged as tutors in the Teaching Certificate course - 38 academic staff and teacher practitioners acquired Teaching Certificate in HPE - students and teaching staff competency assessment - ReFEEHS Introductory symposium organized - ReFEEHS website designed and regularly updated - 5 Project Open-days organized - number of presentations and publication	ReFEEHS Project web site (http://refeehs.com)	- Good communication with non-consortium partner institutions and identification of common goals; - Motivation of healthcare practitioners to be engaged in ExEd as teacher practitioners and their commitment to learning and teaching; - Support from health institutions management - Institutions reluctant to dedicate sufficient amount of their staff working hours to tasks related to students’ professional practice;
		ReFEEHS Needs for Change Report	
**(1) Framework for Experiential Education Development**		ExEd curriculum included in course catalogues at RS HEIs	
		IPE curriculum included in course	
		catalogues at RS HEIs	
		University internal documents–registers/decisions/approvals	
1.1 ExEd competency-based curriculum developed		ReFEEHS Workshops Proceedings	
1.2 QA framework for ExEd developed			
1.3 E-platform for ExEd management and administration designed		VLE educational resources for ExEd, IPE and TCinHPE	
1.4 Educational equipment purchased and installed			
1.5 Reinforced ExEd curriculum implemented		QA Standards for Student Professional Practice Placement in Health Professions Education	
1.6. Students professional competency improved			
**(2) Introduction of interprofessional education**			
2.1 IPE competency-based curriculum developed			
2.2 IPE curriculum implemented			
**(3) Teaching competency development of academic staff and teacher practitioners**		Interprofessional Education Handbook	
3.1 Teaching Certificate in HPE (TCinHPE) course developed			
3.2 Teaching Certificate in HPE course delivered		Teaching & Learning in Health Professions Education–Guide for academic staff	
3.3 Policy for Teaching Competency Development and Evaluation (TCDE)			
3.4 Teaching competency improved			
**(4) Dissemination end Exploitation**			
4.1 ReFEEHS Introductory Symposium			
4.2 Project website designed and maintained			
4.3 Project promotional materials, presentations and publications			
4.4 ReFEEHS Open-days			
4.5 ReFEEHS Final Symposium	ReFEEHS Final Symposium organized		
**Activities**	**Assumptions and risks**		
1.1.1 Identification of ExEd competency-based outcomes	- Awareness of the academic and professional community about the project goals importance; - Dedication of all the HEIs management structures towards the achievement of the project goals; - Students’ acceptance of the new teaching and learning approaches; - Resistance to change from the academic staff and teacher practitioners.		
1.1.2 Developing ExEd educational contents and resources			
1.1.3 Verification of ExEd curriculum updates			
1.2.1 Design and adoption of QA documents for ExEd curricula			
1.2.2 Development of student and teacher practitioner handbooks and guidelines			
1.3.1 Conducting procedure for ICT company subcontracting			
1.3.2 Developing practice sites and teacher practitioners’ databases			
1.3.3 E-platform development			
1.3.4 Exploitation, testing and adaptation of E-platform in practice			
1.4.1 Equipment purchase and installation			
1.5.1 Delivery and evaluation of updated ExEd curriculum			
1.6.1 Evaluation of students’ competency			
2.1.1 Identification of IPE competency-based outcomes			
2.1.2 Developing IPE educational contents and resources			
2.1.3 Verification of IPE curricula			
2.2.1 Pilot implementation and evaluation of new joint IPE courses			
3.1.1 Developing Teaching Certificate curriculum			
3.1.2 Educating tutors for Teaching Certificate in HPE			
3.2.1 Teaching Certificate in HPE course delivery			
3.3.1 Defining Policy for teaching competency development and evaluation			
3.4.1 Evaluation of teaching competency			
4.1.1 Organization of ReFEEHS Introductory Symposium			
4.2.1 ReFEEHS website development and regular update			
4.3.1 Design and printing of ReFEEHS promo-materials, presentations and publications			
4.4.1 Organization of ReFEEHS Open-days			
4.5.1 Organization of ReFEEHS Final Symposium			
